# Feasibility of Detecting Bioorganic Compounds in Enceladus Plumes with the Enceladus Organic Analyzer

**DOI:** 10.1089/ast.2017.1660

**Published:** 2017-09-01

**Authors:** Richard A. Mathies, Md Enayet Razu, Jungkyu Kim, Amanda M. Stockton, Paul Turin, Anna Butterworth

**Affiliations:** ^1^ Department of Chemistry, University of California at Berkeley, Berkeley, California.; ^2^ Department of Mechanical Engineering, Texas Tech University, Lubbock, Texas.; ^3^ Department of Chemistry and Biochemistry, Georgia Institute of Technology, Atlanta, Georgia.; ^4^ Berkeley Space Sciences Lab, University of California at Berkeley, Berkeley, California.

## Abstract

Enceladus presents an excellent opportunity to detect organic molecules that are relevant for habitability as well as bioorganic molecules that provide evidence for extraterrestrial life because Enceladus' plume is composed of material from the subsurface ocean that has a high habitability potential and significant organic content. A primary challenge is to send instruments to Enceladus that can efficiently sample organic molecules in the plume and analyze for the most relevant molecules with the necessary detection limits. To this end, we present the scientific feasibility and engineering design of the Enceladus Organic Analyzer (EOA) that uses a microfluidic capillary electrophoresis system to provide sensitive detection of a wide range of relevant organic molecules, including amines, amino acids, and carboxylic acids, with ppm plume-detection limits (100 p*M* limits of detection). Importantly, the design of a capture plate that effectively gathers plume ice particles at encounter velocities from 200 m/s to 5 km/s is described, and the ice particle impact is modeled to demonstrate that material will be efficiently captured without organic decomposition. While the EOA can also operate on a landed mission, the relative technical ease of a fly-by mission to Enceladus, the possibility to nondestructively capture pristine samples from deep within the Enceladus ocean, plus the high sensitivity of the EOA instrument for molecules of bioorganic relevance for life detection argue for the inclusion of EOA on Enceladus missions. Key Words: Lab-on-a-chip—Organic biomarkers—Life detection—Planetary exploration. Astrobiology 17, 902–912.

## 1. Introduction

Enceladus is an icy moon of Saturn with surface features that range in age from old cratered terrains that formed 4.2–4.6 Ga (Porco *et al.*, 2006; Kirchoff and Schenk, 2009) to the south polar terrain (SPT) dominated by prominent tiger stripe troughs that may be younger than 0.5 Ma (Porco *et al.*, 2006). The high albedo of all terrains (Cruikshank *et al.*, 2005) indicates recent global resurfacing with fresh ice, and it was long suspected that material from Enceladus might contribute to the E-ring of Saturn (Pang *et al.*, 1984). In 2006, Cassini confirmed this suspicion by revealing dozens of distinct, narrow geysers erupting thousands of kilometers into space from four warm fractures in the moon's SPT (Porco *et al.*, 2006, 2014; Spahn *et al.*, 2006; Postberg *et al.*, 2011; Mitchell *et al.*, 2015). This plume has a vapor component that is predominantly water with percent levels of CO_2_, N_2_ or CO (*m/z* = 28), and CH_4_, with trace ammonia and larger light organics such as propane (Waite *et al.*, 2006, 2009). The plume also has a solid particulate component that appears to be stratified in particulate size with the largest salty water particulates being near the surface and smaller micron-scale particulates reaching Cassini fly-by altitudes (Schmidt *et al.*, 2008; Hedman *et al.*, 2009; Schneider *et al.*, 2009; Waite *et al.*, 2009; Postberg *et al.*, 2011; Ingersoll and Ewald, 2011). A recent study, combining measurements from Cassini instruments, estimates the plume column density to be about 3 μL/m^2^ ice at 50 km altitude (Porco *et al.*, 2017, this issue).

Multiple lines of evidence have built a model of Enceladus as having a saline liquid water ocean beneath the icy surface shell that is in direct contact with a rocky core, and that the plume materials are sampled directly from that liquid water ocean (Postberg *et al.*, 2011; Porco *et al.*, 2014; Iess *et al.*, 2014; McKinnon 2015; Thomas *et al.*, 2015). Light organics have been directly detected in the plume emanating from this ocean (Waite *et al.*, 2006, 2009), which may mean this subsurface ocean is enriched with complex organics with *m/z* above the Cassini ion and neutral mass spectrometer (INMS) detection threshold. Indeed, a recent analysis of Cosmic Dust Analyzer data has revealed high mass complex organic cations at the 1% level in 1–3% of the ice particles examined (Postberg *et al.*, 2017).

Analysis of a class of Si-containing, 10 nm scale E-ring particulates indicated ongoing hydrothermal activity at the ocean–core interface (Hsu *et al.*, 2015) similar to that sustained in alkaline hydrothermal vent systems on Earth today. These hydrothermal systems, such as the Lost City system on Earth (Kelley *et al.*, 2005), are alkaline (pH ∼9–11), low-temperature (∼100°C) systems driven by serpentinization and are very different from magmatically heated high-temperature (∼450°C) acidic on-axis systems. The recent observation of molecular hydrogen in the Enceladus vents provides more evidence for deep ocean hydrothermal processes (Waite *et al.*, 2017). Alkaline hydrothermal systems such as those likely on Enceladus have been proposed on Earth to be suboceanic “hot spots” for the emergence of life on Earth (Russell *et al.*, 2010, 2014). In light of the salty liquid water ocean, laced with organic compounds, exhibiting seafloor hydrothermal activity, and venting kilometers into space, Enceladus is a most promising place in the solar system to conduct astrobiological studies.

The most obvious next step in astrobiological analysis at Enceladus is to determine the organic fraction of its plume with highly sensitive and quantitative compositional analysis. The INMS has detected organic species C2–C5 with individual species abundance ∼1 part per thousand (Waite *et al.*, 2011; McKay *et al.*, 2012), but unfortunately can provide little information about organic species with *m/z* above its mass limit (99 Da) or those that do not produce positive ions with high efficiency (Waite *et al.*, 2004). The total organic fraction of the plume could be as high as that found in primitive carbonaceous chondrites; for example, the Murchison meteorite has ∼12 ppm amino acids (Glavin *et al.*, 2010) accumulated through abiotic processes on a primitive parent body that experienced mild heat and liquid water during early solar system formation. Steel *et al.* (2017, this volume) suggest Enceladus has an environment suitable for formation of amino acids both through abiotic processes, after achieving a steady-state ocean amino acid concentration at 0.1 μ*M* levels, and based on availability of H_2_ through serpentinization, they suggest a methanogen-based biotic system that might produce 90 μ*M* total amino acid ocean concentrations.

Amino acids produced abiotically result in a distribution of racemic (or nearly racemic) amino acid speciation in carbonaceous chondrites that is dependent on the weathering and alteration processes that have impacted the extraterrestrial body (Pizzarello *et al.*, 2012; Pizzarello and Yarnes, 2016). Terrestrial life, however, uses only 20 amino acids in its biopolymers, and thus, the terrestrial biosphere is enriched with a biologically focused distribution of homochiral l-amino acids (Lovelock, 1965; McKay, 2004); sea water on Earth has a total amino acid content of roughly micromolar, with individual amino acid concentrations ranging from 1 to 100 n*M*/L (Garrasi *et al.*, 1979). The sensitive, quantitative, compositional, and chiral analysis of amino acids can therefore provide significant information about the multiple processes that have shaped Enceladus, whether they are abiotic or biotic.

To obtain a quantitative, compositional, and chiral analysis of the amino acid composition of the subsurface ocean of Enceladus, multiple requirements must be met. First, the sample must come from that subsurface ocean, which appears to be the case with ice particulate samples of the plume. Capture must occur with high efficiency and with minimal degradation, racemization, and contamination of organic amines and amino acids. Postcapture extraction and analysis must additionally ensure high efficiency with low degradation and racemization; experience with amino acid analysis on Earth and in space has shown that the extraction and analysis systems must both be liquid based. While thermal volatilization gas chromatography/mass spectrometry (GCMS) on Mars, with and without derivatization, has provided information on volatile organic molecules, detection and quantitative compositional analysis of less-volatile oxidized and nitrogen-bearing species of relevance to possible biology-like amino acids remain a challenge for nonliquid-based techniques (Biemann *et al.*, 1976; Stern *et al.*, 2015; Millan *et al.*, 2016). Finally, the detection system must couple the highest possible analytical sensitivity with the liquid-based analysis system. Laser-induced fluorescence (LIF) detection is the most sensitive technique for liquid samples, with concentration limits of detection (LODs) at the detection point as low as 75 p*M* (Chiesl *et al.*, 2009; Stockton *et al.*, 2010). The mass LODs based on the amount of material in a typical 1 nL volume CE injection are 300 zeptomoles, vastly outperforming even the most sensitive GCMS femtogram LODs.

Enceladus may be the best possible target in our solar system for detecting organics relevant for biological habitability and bioorganic compounds that are indicative of past or present life. An efficient capture of pristine ice particulates, followed by liquid extraction, a liquid-based separation, and LIF may be the best combination of tools and techniques with which to characterize the organic content of these deep-sea samples. The Enceladus Organic Analyzer (EOA) is an instrument designed by the Berkeley Space Sciences Laboratory (SSL) based on decades of heritage development at the University of California at Berkeley that has all these attributes. The purpose of this article is to present the EOA instrument concept and proof-of-principle laboratory studies, to present the detailed design and engineering development that have been done toward a flyable instrument, and to present modeling studies establishing the feasibility of successfully capturing and analyzing organic molecules from the Enceladus plume in a fly-by mission profile.

## 2. General Approach and Heritage

**FIG. 1.**
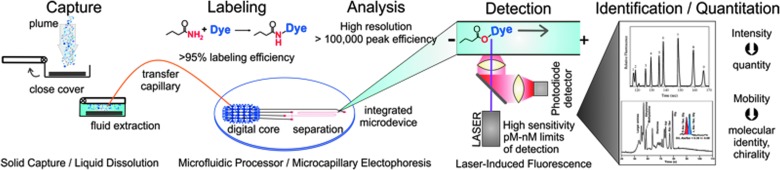
Schematic of the EOA organic plume capture and analysis approach. The top trace presents an analysis of a standard sample of methylamine, ethylamine, citrulline, valine, serine, alanine, glycine, glutamic acids, and aspartic acid (Chiesl *et al.*, 2009). The bottom trace presents an analysis of amino acid composition and chirality from Atacama Desert soil extracts performed in the field with a prototype integrated analyzer instrument (Skelley *et al.*, 2007). EOA, Enceladus Organic Analyzer.

The EOA technical approach for a fly-by instrument is summarized in Figure 1. The instrument can be configured for a single pass fly-by at up to 5–6 km/s or a multiple pass orbiter at lower velocities. The capture door is opened, and the cold ∼160 K exterior capture surface gathers a sample of the Enceladus plume that is ∼10 cm^2^ in cross section and up to 150 km long. After the encounter, the door is closed and, following warming, water solvent is pumped into the chamber to dissolve the captured organic molecules. The chamber is optimized to maximize the collector area and to minimize depth to keep the water volume for dissolution as low as possible. The sample solution is then pumped back to the microfluidic chip where aliquots are mixed with functional group-specific (amine, carboxylic acid, aldehyde/ketone, thiol) fluorescent reagents. The labeled samples are then passed to the microfabricated capillary electrophoresis (CE) system, and high voltage is applied to effect high-resolution separation followed by LIF detection. The fluorescence intensity provides concentration information, and the time of appearance converted to the intrinsic molecular mobility provides molecular identity. EOA can also perform micellar electrokinetic chromatography (MEKC) with a different buffer to provide chiral resolution of amino acids or other chiral species (Chiesel *et al.*, 2009). In this way, part-per-million detection (in the plume particles themselves) of key amine and amino acid and carboxylic acid molecules that inform habitability and potential for or presence of life is achieved. While this description is focused on plume sampling, the core analyzer is equally well suited to organic analysis on a landed instrument, which would not require the plume capture technology described and modeled in this work. If EOA were placed in the plume snow shadow, an open capture plate could capture orders of magnitude more material (Porco et al., 2017, this issue), resulting in part-per-billion detection capability.

The core technologies in the EOA have been extensively developed at Berkeley through the construction and testing of a wide variety of laboratory-based and field instruments and trials over the past 15 years (for a review see Kim *et al.*, 2016). The first organic instrument was the prototype portable Mars Organic Analyzer (MOA), which integrated a microfluidic CE chip with pneumatic valves for solution control, a high-voltage CE channel, and LIF along with electronics for operation in remote environments (Skelley *et al.*, 2005). The MOA prototype was deployed in field trips to the Panoche Valley, California, as well as the Atacama Desert, Chile (Skelley *et al.*, 2005, 2007), where it exploited its 100 p*M* concentration limit of detection to detect amino acids at the low ppb levels in the solid samples. A trace from this work is presented in Figure 1. This work provided the basis for its inclusion in the Urey instrument in Phase A advanced development for the ExoMars rover. This Phase A work led to the development of advanced buffer systems to mitigate the effects of high and low pH samples, high-salt samples, and oxidants (Stockton *et al.*, 2009), low limit of detection labeling and analysis methods with Pacific Blue dye (Chiesl *et al.*, 2009), as well as methods to analyze carboxylic acids, aldehydes, and ketones (Stockton *et al.*, 2010, 2011). This technology also underwent advanced engineering design at the Berkeley SSL for the MOA proposal for Mars 2020.

EOA can analyze for organic amines, carboxylic acids, polycyclic aromatic hydrocarbons (PAHs), etc., to determine organic chemical inventory and likely chemical reactions and processing in the subsurface ocean. Specifically, EOA will analyze for amino acids using amino acid side chain distribution and chirality to probe life indicators and carboxylic acids using chain length variation to probe for biosynthesis (Lovelock, 1965; Balkwill, 1988; McKay, 2004). EOA is significant because its strengths for organic analysis (Stockton *et al.*, 2008, 2009, 2010, 2011) complement the weaknesses of existing techniques (Leshin *et al.*, 2013; Ming *et al.*, 2014). The LIF detection system in EOA provides sub-100 p*M* detection limits in the analyzed solution (Chiesl *et al.*, 2009), and because it is concentration-based rather than mass-based detection it can detect trace (<ppb) species in small micro-to-nanogram input samples (Kaiser *et al.*, 2013). The EOA liquid extraction technique avoids deleterious reactions of organics with oxidants (Stockton *et al.*, 2009) that can compromise thermal volatilization protocols used in GCMS (Freissinet *et al.*, 2015). The EOA prototype was tested on Atacama Desert samples from the Yungay Hills sediments and the Murchison meteorite, and was able to quantitatively determine amino acid composition and chirality from samples (Skelley *et al.*, 2007; Chiesl *et al.*, 2009), where the Sample Analysis at Mars engineering model was unable to detect any organics native to these samples (Stalport *et al.*, 2012). Liquid extraction is also more facile than laser desorption mass spectrometry, which requires that molecules be dissolved in a special matrix with a high laser absorption cross section (Li *et al.*, 2015).

Raman and antibody-based techniques can detect large organic molecules (Parro *et al.*, 2011; Bhartia *et al.*, 2012), but Raman is intrinsically a surface technique for opaque samples with low sensitivity for buried molecules and it requires complex spectral interpretation. Antibody techniques targeted to large, complex biomolecules are Earth-centric approaches (Parro *et al.*, 2011) with nanomolar concentration sensitivity. Antibody techniques that target smaller organic and bioorganic molecules must fortuitously choose the correct targets or develop a very large number of antibody probes. These issues are compounded by the additional difficult requirement for long-duration stabilization of sensitive antibody proteins (Diego-Castilla *et al.*, 2011).

## 3. Capture Chamber Design and Rationale

**FIG. 2.**
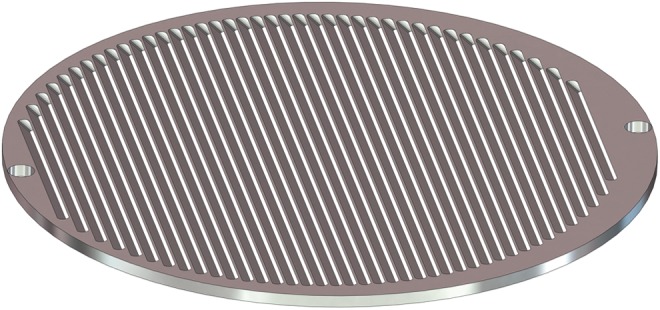
CAD of the tilted louver particle capture structure in EOA. The louvers are thin 1100 Al slats that capture half of the incoming particles and permit the other half to pass through to the underlying 1100 Al chamber surface. CAD, computer-aided design.

The tilted louver structure at the input face of the capture chamber is presented in Figure 2. The surfaces that are impacted by the incoming ice particles are fabricated of soft 1100 alloy aluminum to direct the particle kinetic energy into target deformation and to provide efficient thermal transfer of the impact heating into the relatively massive Al surface. These surfaces are also readily cleaned of organic residues and contaminants via oxidative pyrolysis and other standard cleaning protocols.

The EOA capture chamber design is justified by the observation that the capture and survival of organic compounds in high-velocity encounters from 200 m/s to 6 km/s have been demonstrated. U2 aircraft routinely captures interplanetary dust particles with significant organic matter at 200 m/s (Brownlee, 1985). Glavin *et al.* (2008) and Elsila *et al.* (2009) recovered nanomole amounts of cometary methyl amine, ethyl amine, glycine, and likely β-alanine from Stardust 1100 foils following 6.1 km/s impacts. Burchell *et al.* (2014) and Bowden *et al.* (2009) impacted organic-containing ice with projectiles at up to 4.9 km/s and demonstrated significant recovery of labile β-carotene as well as stearic acid and anthracene. Thus, significant data support the survival and capture of organic compounds in hypervelocity impacts with amounts readily detectable by EOA.

**FIG. 3.**
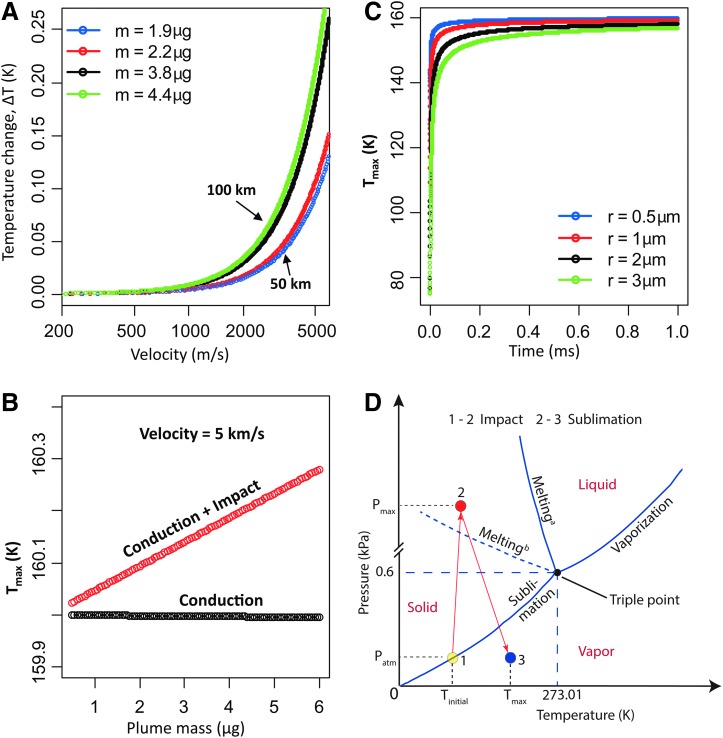
Theoretical modeling of high-velocity ice particle collection on 1100 Al foil. **(A)** Profile of the temperature change (ΔT) in the captured ice and foil due to impact of four different total captured plume masses. Plume mass was calculated for an altitude of 50 km for 50 km and 100 km plume width from Visual and Infrared Mapping Spectrometer data for both masked and unmasked capture chamber diameter as shown in Supplementary Data. **(B)** Maximum temperature of the ice plume and fly-by capture chamber at 5 km/s impact velocity as a function of plume mass for conduction only and for the combined conduction and impact case, which is slightly above the foil temperature. **(C)** Transient temperature profile of individual ice particles versus time after impact using transient heat conduction for semi-infinite solid as in Equation 4 in Supplementary Data. **(D)** Pressure–Temperature (P–T) diagram for water illustrating the possible thermodynamic state of collected ice based on temperature analysis in **(A–C)** and the pressure in space and pressure after impact for an individual particle using Hooke's law and the elastic modulus of 1100 Al. Minimum pressure (P_min_) is unknown, P_max_ is 40 GPa, T_initial_ is 75 K, and T_max_ is 160.2 K. Yellow, red, and blue dots show the three states of the ice particle—initial, after impact, and before closing the door, respectively. The liquid-solid coexistence curve is indicated under static pressure conditions (Melting^a^) and also shock pressure conditions (Melting^b^).

In more detail, the capture chamber design consists of a louvered screen placed over a shallow capture chamber with an overall impact area of 10 cm^2^. The louver screen consists of a parallel array of 100 micron thick louvers fabricated of 1100 Al foil tilted at an angle of 45° with a size and spacing chosen such that 50% of the incoming particles strike the louvers and 50% pass through to the 1100 Al surface below. This design ensures efficient capture of any material that rebounds from the underlying surface impact. The ice particles will strike the cold soft Al surface where the majority of the kinetic energy is deposited into the target in the form of target deformation and heat transfer to the target (Melosh, 1989 and Figure 3).

The kinetic energy that remains will heat and potentially melt the ice. Any material that vaporizes on impact will produce a water organic molecular plume that condenses on the interior of the chamber and the backside of the louvers. Multiple passes can be made to add additional sample, and after closing the collector lid sealing the extraction chamber, the warmed capture plate is washed to remove collected organics for analysis.

If the Enceladus ocean sample harbors extant organisms such as bacteria adapted to extreme environments, impacts in the 2–5 km/s range should produce significant shock and/or heat lysis of cells destroying physical cellular structure but releasing their more stable chemical components and metabolites for chemical analysis. For impacts that do not shatter the cells (Burchell *et al.*, 2001; Horneck *et al.*, 2006), we still expect efficient cell lysis due to the 25 atm difference in osmotic pressure between our water buffer solution and the cell interior, which would be adapted to a very salty Enceladus ocean environment.

A second important consideration is the density of the plume and the amount of material that can be captured in one transit. The plume density estimates vary as a function of plume width, pass altitude, and time. By using the recent estimated plume column density of about 3 μL/m^2^ at 50 km altitude (Porco *et al.*, 2017, this issue), one transect of a 10 cm^2^ area capture chamber would thus encounter 3 μg ice, which, assuming 1 ppm levels, captures 3 pg of amino acids. Steel *et al.* (2017, this volume) suggest that amino acids (glycine, alanine, AIB, and glutamic acid) in the ambient Enceladus ocean could total 0.4 μ*M* due to abiotic production, which corresponds to ∼0.1 pg of amino acids in 3 μg captured ice. Their modeled methanogenic amino acid production result was 90 μ*M* ocean and plume amino acid concentration, which would result in 20 pg total amino acids collected in a single 10 cm^2^ transect. A 90 μ*M* collected sample is equivalent to a 2000 p*M* CE injection, which is easily detectable given the high sensitivity of the EOA instrument.

## 4. Numerical Analysis of Ice Particle Impact Temperature

The ice particle impact temperature profile is important for determining the ice capture efficiency as well as the likelihood of organic analyte survival. From conservation of momentum and energy as shown in Equations 1 and 2 in the Supplementary Data (Supplementary Data are available online at www.liebertonline.com/ast), the change in kinetic energy of the incoming ice plume due to foil impact results in heating of the ice and the collection chamber foil, deformation of the foil, and vibration of the chamber. In this modeling, we make a worst-case assumption that 50% of the kinetic energy is converted to heating of the captured material and the soft Al 1100 target (Melosh, 1989). Numerical analysis indicates only a negligible increase (0.25 K) in the final temperature of the collected ice plume and chamber due to plume impact for the collection of 4.4 μg ice corresponding to the unmasked capture chamber diameter and 100 km plume width for an altitude of 50 km as shown in Figure 3A calculated from Equation 3 in the Supplementary Data.

The maximum temperature of the ice and foil after the impact was calculated for conductive heat transfer and the combination of impact and conduction from Equation 5 in the Supplementary Data under the assumption of minimum radiative heat gain/loss from space to the foil for the period of ice particle collection (Fig. 3B). The maximum temperature remains in a range of 159.98–160.25 K, which is close to the initial temperature of the foil with negligible increase due to impact for the range of plume mass captured.

The surface temporal profile of the ice projectile temperature during impact was also calculated for individual impacting particles of 0.5, 1, 2, and 3 μm radius using Equation 4 in the Supplementary Data (Fig. 3C). The temperature of the ice particle exponentially increases to 120–140 K in a few nanoseconds, while asymptotically reaching the maximum contact surface temperature (160.25 K) in 100 ns to 1 ms with slight variation for varying particle sizes (0.5–3 μm), respectively. With this temperature profile, no thermal degradation of entrained organics is expected.

A phase change of the ice particle from solid to liquid and especially vaporization should be avoided for efficient capture of organic molecules in the incoming ice. The phase change depends on the temperature and pressure of the ice particle during and after impact; it is desirable to remain to the left and above the triple point in Figure 3D (0.6 kPa, 273.01 K). Since the Enceladus plume consists of a mixture of ice particles and vapor, there is a possibility that the initial thermodynamic state of the captured ice particles is a quasiequilibrium between the water ice and vapor. During the impact, the pressure and temperature in the ice particle increase momentarily (1–2 in Fig. 3D) and then the pressure starts to drop, whereas temperature keeps increasing up to 162.25 K. At the final state indicated by 3 in Figure 3D, there is likely sublimation of the water in the captured ice due to elevated temperature and low pressure before the capture door is closed.

## 5. EOA Design Schematics and Operation

**FIG. 4.**
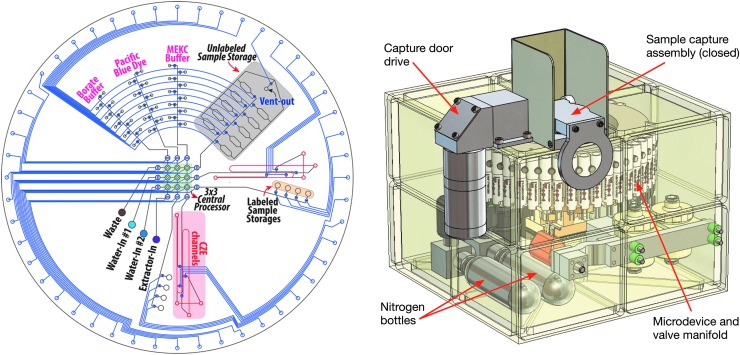
CAD of the EOA chip fluidic processor and integrated instrument. The TRL of the microfluidic valve array, the microfluidic CE system, and the LIF system is 5; the TRL of the capture plate is currently 3 so this will be the primary focus of development work in the near future along with subsystem integration. CE, capillary electrophoresis; LIF, laser-induced fluorescence; MEKC, micellar electrokinetic chromatography; TRL, technology readiness level.

A computer-aided design of the entire EOA instrument along with a mask design for the microchip analyzer is presented in Figure 4. The overall instrument is a compact 2.5 kg in a 16 × 16 × 12 cm package with low 2–3 watt survival power requirements to stay between −10°C and −50°C. The capture chamber design, operation, and functional parameters and an overview of EOA operation were already described. Below, we detail the operational process and parameters.

Macroscopic fluid transport between major components of the instrument such as the extractant chamber, capture chamber, and waste reservoirs is driven by applying gas pressure through a stainless steel bellows system. All macroscopic volumes of fluids are contained within a welded stainless steel bellows that can be expanded or contracted by externally applied gas pressure. This approach avoids the problem of gas-fluid bubbles in large containers under microgravity.

Fluidic transport in microchannels is driven by pneumatic peristaltic pumps fabricated within the chip itself via typically three polydimethylsiloxane (PDMS) membrane valves placed in series (Grover *et al.*, 2003). In the 50–500 micron features within the chip, water surface tension is the dominant force compared to gravity, and pumped liquids reliably sweep bubbles out of the microfluidic channels. These pumps and channels work well right side up (+1 g force) and upside down (−1 g force) and so reliable operation in microgravity is anticipated (Stockton *et al.*, 2016). PDMS is stable well outside the anticipated operational parameters, and its decomposition products include glass and other species that do not interfere with EOA analyses.

Figure 4 presents the design of the 100 mm diameter PMA microdevice showing both the PDMS multilayer pneumatic features (blue), PDMS fluidic channels (black), and the glass features (red) in projection. The features on the perimeter indicate where the 48 pneumatic inputs pass into the PMA from the manifold solenoids to control fluidic processing by valve actuation. The two 30 × 100 micron by 10 cm long glass CE channels are fabricated within a thermally fused glass sandwich structure drilled via holes that fluidically connect the four ends of the CE system with the PDMS fluidic layers. The central core consists of a 3 × 3 valve array processor with each perimeter valve of the array controlled by a gate valve. Each leg of the CE system (sample, waste, cathode, and anode) is connected to the central processor by a fluidic channel that is controlled to route fluid to the desired CE leg. Two redundant CE systems are presented in this design.

There are four primary inputs to the PMA that connect with the capture chamber, the two primary solvent reservoirs, and the waste. The 90 reagents and buffers needed for 8 assay series are placed dry in the radial features at the top of the chip. These chambers are addressed by a binary coding format where a reagent is selectively hydrated by pneumatically addressing one of the circumferential lines while pumping water up a selected radial from the 3 × 3 processor. In this way, only 19 control valves are needed to address 90 different reagent reservoirs.

To perform an analysis, the closed capture chamber containing sample is warmed and buffer is transported into the chamber dissolving the sample. The dissolved sample is then transported from the capture reservoir to the storage location on the chip (Kim *et al.*, 2016). The amine labeling and analysis are representative; analogous methods for analyzing organic acids (Stockton *et al.*, 2011), aldehydes and ketones (Stockton *et al.*, 2010), thiols (Mora *et al.*, 2013), and PAHs (Stockton *et al.*, 2008) have been presented. For amine analysis, water is first pumped to one of the dry Pacific Blue storage reservoirs to dissolve and mobilize the labeling reagent, and water is pumped to one of the pH 9.5 borate storage reservoirs to hydrate the labeling buffer. Then, alternating 500 nL aliquots of the Pacific blue sample and labeling buffer are pumped by the processor to one of the labeled sample storage locations for a 5–10 min incubation. The pH 9.5 buffer is pumped to the CE reservoirs where the channels fill rapidly by capillary action. The reacted sample is then diluted with separation buffer and pumped to the CE reservoir for injection and analysis. To perform additional separations of similarly sized amines and for chiral analysis of amino acids, the stored Pacific Blue-labeled sample is mixed with the hydrated CHAPSO (3-([3-cholamidopropyl] dimethylammonio)-2-hydroxy-1-propane sulfonate) detergent buffer by the PMA and processed for chiral MEKC analysis (Chiesl *et al.*, 2009). After separation, the fluid in the CE reservoirs is pumped out by the processor and sent to waste, and the channel is washed to remove residual buffer components.

The electrophoretic analysis process consists of loading the sample into the CE cross by applying 500 V/cm from sample to waste for 30 s. Then, a field of 600 V/cm is applied to the separation channel inducing electro-osmotic flow. The molecules in the sample are swept by the electro-osmotic flow toward the detector but also experience a charge-based force and a size-based drag, providing the unique molecular charge and size information that determine identity. Typical separations take <5 min. If analyte concentrations are high, digital dilutions can be performed in the PMA followed by reanalysis. If the sample concentrations are low, a stack or direct injection can be performed for 2–10 s to increase the amount of sample injected by more than 10-fold and extend the detection limits. The detection system will use a 405 nm diode laser focused to a 30 micron spot within the CE channel and emission gathered by a high numerical aperture lens and passed through a long-pass filter and confocal filter to a photodiode.

**FIG. 5.**
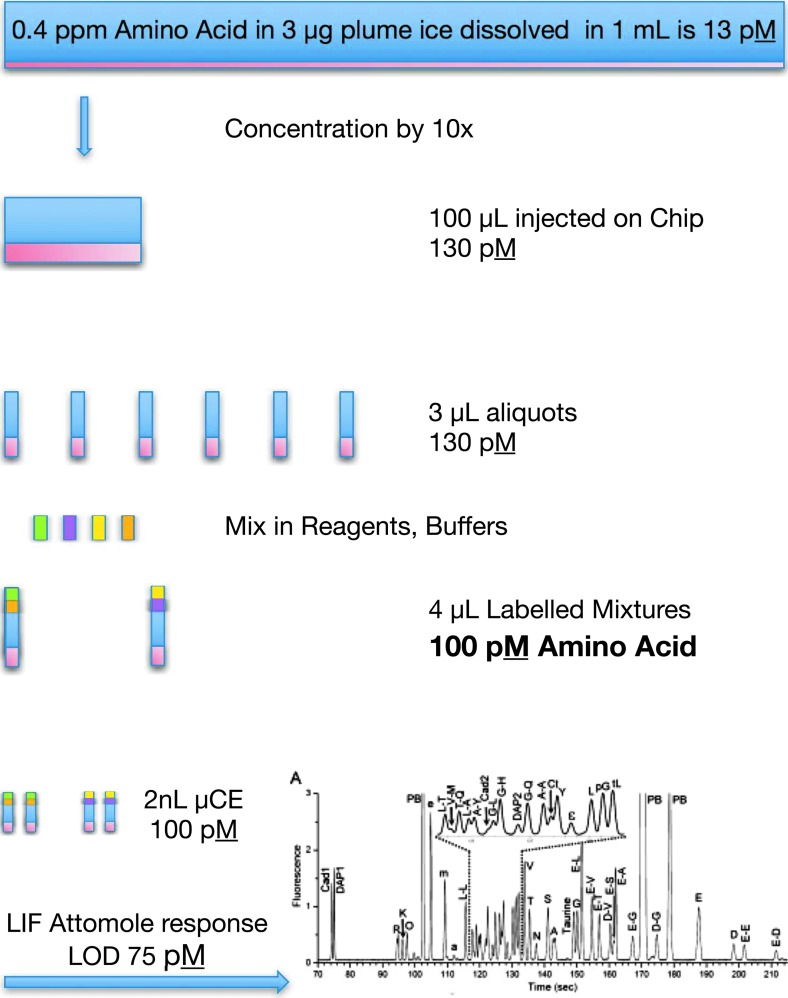
Outline of transfer process for the serial solution transfers in the EOA. Based on the 75 p*M* limit of detection at the LIF detector, the EOA should be able to detect as little as 100 p*M* amino acid, which translates to only 0.4 ppm amino acid in an assumed 3 μg of captured ice. Thus, the EOA has the capability to detect the capture of as little as 1 pg of an organic species in the capture chamber. The bottom trace presents a microchip CE analysis of a complex mixture of organic amines and amino acids demonstrating the high-resolution rapid separations with 75 p*M* limits of detection that are achieved with Pacific Blue labeling (Chiesl *et al.*, 2009).

With the optimized dye Pacific Blue, the detection limit for normal injection is 75 p*M* (Chiesl, 2009), but this limit can be improved ∼10-fold by stack injection to 10 p*M*. This is equivalent to the detection of only attomoles of sample material. A typical example of a CE analysis trace of labeled amines from Chiesl *et al.* (2009) is presented in Figure 5.

The analysis process and expected performance of the EOA are outlined in Figure 5. Based on the 75 p*M* limit of detection at the LIF detector, the EOA should be able to detect as little as 100 p*M* amino acid, which translates to only 0.4 ppm amino acid (only 1 pg) in an assumed 3 μg of captured ice. The EOA picomolar limit of detection and microfluidic fluid handling enables the split of the 100 μL concentrated sample into multiple aliquots while maintaining concentrations at a high enough ratio to the limit of detection to enable quantitation. This LOD is much better than the nanomolar LODs of the best antibody-based analysis systems. In addition, EOA can perform spiking experiments of known standards to reduce ambiguity of identification, since each analysis requires in total only a few microliters of labeled sample solution. While the radiation resistance of the Pacific Blue and Cascade Blue fluorescent probes and of the buffers used by EOA has not yet been explicitly determined, similar fluorescent probes, including fluorescein, SYBR Green, and Alexa Fluor 633, all show more than sufficient stability for a mission profile like that of the EOA (Thompson *et al.*, 2006; Le Postollec *et al.*, 2009; de Diego-Castilla *et al.*, 2011; Carr *et al.*, 2013). Furthermore, the dyes and buffers are stored dry, cold, and in an inert atmosphere on-chip to minimize chemical degradation.

We also consider the performance of the EOA instrument if the amount of ice captured is <3 μg. For example, Porco et al. have estimated that the plume ice density may range from 0.2 to 6 μL/m^2^ (Porco *et al.*, 2017, this issue), resulting in the capture of 0.2–6 μg of ice by our system; achieving a ppm limit of detection could be challenging if <1 μg ice is captured. One solution to this problem is to perform multiple passes through the plume with an orbiter at a lower altitude to gather more material. In this circumstance, the capture chamber door is open for each pass, and the sample is integrated on the cold capture chamber surface before analysis. An additional way to get more sample would be to shift to a landed configuration and gather plume “snow” with an open capture chamber (Porco *et al.*, 2017, this issue). Second, given that the solution volume returned from the capture chamber (1000 μL) is much more than that needed for microfluidic analysis, additional concentration (∼10-fold) can be performed on-chip to compensate for the reduced sample mass. Third, we can perform stack injection on the CE system to decrease the limit of detection by a factor of 10 and accept a modest loss of resolution to maintain the ppm limit of detection. Thus, the EOA has the capability to detect ppm organic molecules even if the plume density is at its lower estimated limits.

## 6. Discussion and Conclusions

We have presented the concept as well as a practical design for the EOA that can gather ice particles from an Enceladus plume and sensitively analyze for organic molecules, especially organic amines and amino acids that are indicative of habitability and potentially life. In addition, calculations are presented for the amount of ice that can be captured in a single plume transect based on latest available plume density estimates, and the impact is modeled to determine the nature of the impact and the maximum sample temperature during capture. Enceladus plume density understanding is still in flux, but EOA can detect specific organic species at the ppm level from only micrograms of captured ice because of its picogram organic mass detection limits. The organic content in the buried ocean may be lower than parts per million; for example, the 0.1 μ*M* (0.01 ppm) abiotic amino acid oceanic concentration suggested by Steel *et al.* (2017) would result in EOA collection of a 30 fg amino acid sample. However, formation of the plume itself induces bubble scrubbing of organics off surfaces that can enhance the organic content by factors of 100–1000 (Porco *et al.*, 2017, this volume). This enhancement would allow even the lowest expected amino acid concentration to be analyzed by EOA.

Thermal modeling shows that the ice particles that impact the soft 1100 Al target with a velocity from 200 m/s to 5 km/s melt as a molecular solid in static pressure (Melting^a^ in Fig. 3D) and as a liquid in shock pressure (Melting^b^ in Fig. 3D), and their temperature rises to the ambient temperature of the target 160 K on a millisecond time scale (Stewart and Ahrens, 2003; Schwegler *et al.*, 2008). This modest heating is due to the small mass-driven kinetic energy of the incoming particles and the rapid thermal equilibration between the ice particle (with a high surface to volume ratio) and the aluminum surface in which it is imbedded. This thermal profile supports the efficient capture of incoming ice particles because they do not melt or vaporize on impact. This profile also supports the idea that any organic molecules in the ice will not be degraded on impact and that any amino acids or other bioorganics will not be isomerized or racemized thereby preserving composition and chirality as a probe for extraterrestrial life.

The environmental challenges associated with flying a microfluidic system in space are also clearly surmountable. Multilayer microfluidic chips with valves were first made in 2003 and found to survive many thousands of cycles (Grover *et al.*, 2003). Chips made for the MOA in 2005 were recently examined and found to be fully functional after 10 years of storage (Duca *et al.*, 2015; Stockton *et al.*, 2016). Thus, the microfluidic systems and valve arrays are quite robust. Because the instrument is designed to house the microdevice in a gas-pressurized chamber, operation of the instrument in the vacuum of space should not present a problem. Microfluidic operation in a microgravity environment is also not expected to present an issue because surface tension of fluids in the internal micron-sized channels is the dominant force that controls fluid behavior. Bubbles should not form in the microfluidic systems because of the high surface tension of water, but if formed do not significantly impact performance (Stockton *et al.*, 2016) and can be eliminated by the use of degassing membranes. Finally, fluidic leaks and contamination are unlikely because the entire microfluidic system is monolithically integrated (as opposed to troublesome tubing systems assembled from components); furthermore, if leaks do occur, the volumes of material are very small—on the microliter scale. We conclude that microfluidics presents a uniquely capable approach for performing complex processing of liquid samples for many types of analyses in flight applications.

Once developed, EOA will provide an important expansion of National Aeronautics and Space Administration (NASA) technical capability for the analysis of plume, surface, or subsurface samples from Enceladus, Europa, Titan, or comets. While landed missions generally sample more material, one significant advantage of the fly-by configuration is that the EOA instrument does not require stringent planetary protection. Such missions with EOA technical capability should answer key Decadal Survey questions such as “are there modern habitats elsewhere in the solar system with necessary conditions, organic matter, water, energy, and nutrients to sustain life, and do organisms live there now?”

## Supplementary Material

Supplemental data

## Abbreviations Used

CAD: computer-aided design
CE: capillary electrophoresis
EOA: Enceladus Organic Analyzer
GCMS: gas chromatography/mass spectrometry
INMS: ion and neutral mass spectrometer
LIF: laser-induced fluorescence
LODs: limits of detection
MEKC: micellar electrokinetic chromatography
MOA: Mars Organic Analyzer
PAHs: polycyclic aromatic hydrocarbons
PDMS: polydimethylsiloxane
SPT: south polar terrain
SSL: Space Sciences Laboratory
