# Supplementary material for: Feasibility of Detecting Bioorganic Compounds in Enceladus Plumes with the Enceladus Organic Analyzer
Source: Astrobiology. 2017 Sep 1;17(9):902–12. doi: 10.1089/ast.2017.1660 (PMC5610425; doi:10.1089/ast.2017.1660)
Supplement: Supplemental data [file Supp_Data.pdf]

Supplementary Data

Table 1: Properties of ice particle and 1100 alloy aluminum foil

	Density (kg/m^3)	Initial temperature (K)	Conductivity, k W/mK	Heat capacity, c J/kg-K
Ice particle	934	75	3.48	2100
Alloy Al 1100 foil	2710	160	218	900
Foil thickness = 100 µm[3], Foil area (Capture area) = 9.9 – 11.4 cm^2				
Ambient Pressure = unknown, but likely very thin				

Mathematical equations and analysis (Momentum and kinetic energy conservation takes into account the mass of whole flyby and mass of ice plume.)

Conservation of momentum

$$m_{flyby} u_{flyby} + m_{iceplume} u_{iceplume} = (m_{flyby} + m_{iceplume}) v \qquad (1)$$

Where v is the combined final velocity of flyby and ice particle

$$v = \frac{m_{flyby} u_{flyby} + m_{iceplume} u_{iceplume}}{m_{flyby} + m_{iceplume}}$$

$$v = u_{flyby} + \frac{m_{iceplume}}{m_{flyby}} u_{iceplume}$$ Since $m_{iceplume} << m_{flyby}$

$$v = u_{flyby}$$ Since $m_{iceplume} / m_{flyby} \sim 0$

Conservation of kinetic energy

Initial kinetic energy

$$\frac{1}{2} m_{flyby} u_{flyby}^2 + \frac{1}{2} m_{iceplume} u_{iceplume}^2$$

Final kinetic energy

$$\frac{1}{2}(m_{flyby} + m_{iceplume}) v^2 \cong \frac{1}{2} m_{flyby} u^2{}_{flyby}$$ Since $v = u_{flyby}$ from momentum conservation in equation 1

Hence, kinetic energy loss = Initial kinetic energy – Final kinetic energy = $\frac{1}{2} m_{iceplume} u_{iceplume}^2$ (2)

*Thermodynamic and heat transfer analysis takes into account the mass of foil and mass of ice particle

Increase in temperature of the ice particle and foil

50% of the kinetic energy converts to heat

Lost kinetic energy = $\text{Heat}_{icepartcle}$ + Heat_{foil}

$$50\% \text{ of } \frac{1}{2} m_{iceplume} u_{iceplume}^2 = m_{iceplume} c_{iceplume} \Delta T + m_{foil} c_{foil} \Delta T \qquad (3)$$

$$\Delta T = \frac{\frac{1}{4} m_{iceplume} u_{iceplume}^2}{m_{iceplume} c_{iceplume} + m_{foil} c_{foil}}$$

Profile of maximum temperature with respect to time

Assumptions: Transient heat conduction for semi-infinite solid/ Temperature profile of ice plume

$$\frac{T(x,t) - T_i}{T_s - T_i} = erfc(\frac{x}{2\sqrt{\alpha t}}) \qquad (4)$$

where T_i is the initial temperature of the ice plume, T_s is the equilibrium surface temperature, t is the time, α is the thermal diffusivity defined by k/ρc of the ice, x is the distance from the contact surface.

Equilibrium temperature (Maximum temperature) of ice plume and foil

$$m_f c_f (T_f - T_{\max}) = m_{iceplume} c_{iceplume} (T_{\max} - T_i) \qquad (5)$$

**Table 2:** Ice collection per pass for different plume widths and collection chamber diameters			
	Collector chamber area (cm2)	Ice collection per pass (µg)	
		Plume width 50 km	Plume width 100 km
Masked	9.9	1.917343	3.834687
No mask	11.4	2.20785	4.4157
Altitude = 50 km, Density of ice = 0.934 g/cm^3, Particle sizes 1, 2, and 3 µm with number density from VIMS [4]			

REFERENCES

[1] M. Peplow, "Atmosphere found on Enceladus," *Nature, ,* doi:10.1038/news050314-15, 2005.

[2] S. W. Kieffer, X. Lu, C. M. Bethke, J. R. Spencer, S. Marshak, and A. Navrotsky, "A Clathrate Reservoir Hypothesis for Enceladus' South Polar Plume," *Science,* vol. 314, pp. 1764-1766, 2006.

[3] M. C. Price, A. T. Kearsley, M. J. Burchell, L. E. Howard, J. K. Hillier, N. A. Starkey*, et al.*, "Stardust interstellar dust calibration: Hydrocode modeling of impacts on Al-1100 foil at velocities up to 300 km s−1 and validation with experimental data," *Meteoritics & Planetary Science,* vol. 47, pp. 684-695, 2012.

[4] M. M. Hedman, P. D. Nicholson, M. R. Showalter, R. H. Brown, B. J. Buratti, and R. N. Clark, "Spectral Observations of the Enceladus Plume with Cassini-VIMS," *The Astrophysical Journal,* vol. 693, p. 1749, 2009.
